# Supplementary material for: Effects of Exercise Combined with a Healthy Diet or Calanus finmarchicus Oil Supplementation on Body Composition and Metabolic Markers—A Pilot Study
Source: Nutrients. 2020 Jul 18;12(7):2139. doi: 10.3390/nu12072139 (PMC7400904; doi:10.3390/nu12072139)
Supplement: Supplementary file 1 [file nutrients-12-02139-s001.zip › nutrients-830982-supplementary.docx]

**Table S1.** Questionnaire based physical activity levels of the participants at baseline (0), after six weeks (6) and 12 weeks after the intervention (12).

| **Parameters** |  | **CON** | **EX** | **EXDC** | **EXCO** | ***p*** |
| --- | --- | --- | --- | --- | --- | --- |
|  | **t** |  |  |  |  |  |
| Basal activity | 0 | 4.26 ± 3.81 | 4.17 ± 6.02 | 2.14 ± 1.86 | 3.12 ± 2.85 | 0.257 |
|  | 6 | 4.84 ± 4.70 | 4.50 ± 3.76 | 2.95 ± 2.51 | 5.18 ± 4.34 |  |
|  | 12 | 6.73 ± 10.0 | 6.80 ± 8.97 | 4.99 ± 4.31 | 6.66 ± 5.13 |  |
| Leisure time activity | 0 | 3.04 ± 3.95 | 2.14 ± 2.74 | 1.16 ± 1.10 | 1.90 ± 2.26 | 0.184 |
|  | 6 | 3.69 ± 4.66 | 2.39 ± 2.20 | 2.67 ± 2.00 | 1.71 ± 2.28 |  |
|  | 12 | 4.36 ± 6.46 | 3.89 ± 5.59 | 2.65 ± 2.59 | 3.22 ± 4.48 |  |
| Sport activity | 0 | 0.38 ± 1.04 | 0.33 ± 0.52 | 0.30 ± 0.69 | 0.31 ± 0.95 | 0.468 |
|  | 6 | 0.40 ± 0.90 | 0.54 ± 1.21 | 0.32 ± 0.71 | 0.53 ± 0.85 |  |
|  | 12 | 0.20 ± 0.47 | 0.50 ± 0.95 | 0.32 ± 0.70 | 0.19 ± 0.46 |  |
| Total activity (hours/week) | 0 | 7.69 ± 5.34 | 6.63 ± 7.60 | 3.59 ± 2.56 | 5.33 ± 3.88 | 0.151 |
|  | 6 | 8.93 ± 7.37 | 7.43 ± 5.66 | 5.94 ± 3.94 | 7.42 ± 4.80 |  |
|  | 12 | 11.3 ± 14.5 | 11.2 ± 12.2 | 7.97 ± 6.02 | 10.1 ± 7.0 |  |

Data shown as mean ± SD. Data were analyzed using two way repeated measure ANOVA. P values represent time*intervention interaction. t=time in weeks.

**Table S2.** Dietary Intake of food groups at baseline (0), after six weeks (6) and at the end of the.

intervention (12).

| **Parameters** |  | **CON** | **EX** | **EXDC** | **EXCO** | ***p*** |
| --- | --- | --- | --- | --- | --- | --- |
|  | **t** |  |  |  |  |  |
| Fruit intake (portion/day) | 0 | 1.39 ± 1.06 | 1.85 ± 1.09 | 1.43 ± 0.98 | 2.02 ± 2.48 | **0.006** |
|  | 6 | 1.27 ± 0.92 | 1.78 ± 1.42 | 1.96 ± 0.96 | 1.78 ± 1.47 |  |
|  | 12 | 1.32 ± 0.78 | 1.83 ± 1.89 | 2.14 ± 1.26**‡** | 1.86 ± 1.52 |  |
| Vegetable intake (portion/day) | 0 | 1.32 ± 1.87 | 1.10 ± 0.79 | 0.86 ± 0.34 | 0.95 ± 0.76 | **0.001** |
|  | 6 | 0.95 ± 0.63 | 0.97 ± 0.70 | 1.17 ± 0.74 | 0.97 ± 0.59 |  |
|  | 12 | 0.77 ± 0.48***** | 1.10 ± 0.97 | 1.49 ± 1.25**‡** | 0.96 ± 0.73 |  |
| Meat intake (portion/day) | 0 | 1.63 ± 1.63 | 2.02 ± 2.04 | 1.33 ± 1.07 | 1.57 ± 1.18 | 0.480 |
|  | 6 | 1.77 ± 2.31 | 1.57 ± 1.68 | 1.08 ± 0.77 | 1.08 ± 0.79 |  |
|  | 12 | 1.51 ± 1.63 | 1.41 ± 1.45 | 1.06 ± 0.85 | 1.08 ± 0.88 |  |
| Meat intake (g/week) | 0 | 621.9 ± 333.2 | 667.0 ± 494.9 | 528.81± 346.6 | 523.2 ± 268.3 | 0.225 |
|  | 6 | 605.1 ± 384.2 | 485.3± 385.6 | 443.82± 256.3 | 383.4 ± 231.0 |  |
|  | 12 | 536.4 ± 322.8 | 504.8 ± 327.1 | 418.1± 258.0 | 383.8 ± 207.9 |  |
| Cereal intake (portion/day) | 0 | 3.38 ± 1.97 | 3.38 ± 2.37 | 3.03 ± 1.77 | 3.17 ± 1.44 | 0.521 |
|  | 6 | 2.80 ± 1.71 | 2.81 ± 1.56 | 2.67 ± 1.03 | 3.28 ± 1.85 |  |
|  | 12 | 2.52 ± 1.06**†** | 2.73 ± 1.55 | 2.75 ± 1.26 | 2.84 ± 1.30 |  |
| Milk intake (portion/day) | 0 | 2.35± 1.80 | 3.02± 1.82 | 3.22± 2.15 | 3.01± 2.32 | 0.366 |
|  | 6 | 2.22± 1.62 | 2.51± 1.41 | 3.20± 1.07 | 2.83± 2.39 |  |
|  | 12 | 2.08± 1.48 | 2.51± 1.56 | 3.71± 2.47 | 2.91± 2.03 |  |
| Fish intake (portion/week) | 0 | 1.23± 1.10 | 1.49± 2.18 | 1.77± 1.53 | 1.26± 1.05 | **0.010** |
|  | 6 | 1.28± 0.99 | 1.16± 1.00 | 2.51± 1.94 | 1.70± 1.53 |  |
|  | 12 | 1.08± 0.72 | 1.23± 1.06 | 2.69± 1.71**‡** | 1.36± 1.04 |  |

Data shown as mean ± SD. P values represent time*intervention interaction analyzed with two way repeated measure ANOVA. In case of significance, asterisks indicate statistical differences within groups detected with post hoc Bonferroni (* *p* < 0.05; † *p* < 0.01; ‡ *p* < 0.001).t=time in weeks.
